# Why war is a man's game

**DOI:** 10.1098/rspb.2018.0975

**Published:** 2018-08-15

**Authors:** Alberto J. C. Micheletti, Graeme D. Ruxton, Andy Gardner

**Affiliations:** School of Biology, University of St Andrews, Dyers Brae, St Andrews KY16 9TH, UK

**Keywords:** war, violence, sex differences, competition, hysteresis, behavioural disorders

## Abstract

Interest in the evolutionary origins and drivers of warfare in ancient and contemporary small-scale human societies has greatly increased in the last decade, and has been particularly spurred by exciting archaeological discoveries that suggest our ancestors led more violent lives than previously documented. However, the striking observation that warfare is an almost-exclusively male activity remains unexplained. Three general hypotheses have been proposed, concerning greater male effectiveness in warfare, lower male costs, and patrilocality. But while each of these factors might explain why warfare is more common in men, they do not convincingly explain why women almost never participate. Here, we develop a mathematical model to formally assess these hypotheses. Surprisingly, we find that exclusively male warfare may evolve even in the absence of any such sex differences, though sex biases in these parameters can make this evolutionary outcome more likely. The qualitative observation that participation in warfare is almost exclusive to one sex is ultimately explained by the fundamentally sex-specific nature of Darwinian competition—in fitness terms, men compete with men and women with women. These results reveal a potentially key role for ancestral conditions in shaping our species' patterns of sexual division of labour and violence-related adaptations and behavioural disorders.

## 1. Introduction

Recent contributions from multiple disciplines—including archaeology, psychology, evolutionary biology, and anthropology—have greatly deepened our understanding of warfare, which may be broadly defined as coalitionary intergroup aggression [1–28]. However, the extreme sex difference in individuals' involvement in warfare remains unexplained. In our evolutionary past, warfare was mainly—most likely, almost-exclusively—a male pursuit, as revealed by major discoveries of prehistoric mass graves and other material evidence of lethal intergroup conflict [24,26,27,29,30]. Similarly, in the vast majority of historical and contemporary hunter–gatherer and small-scale societies, women have only rarely participated in warfare in a direct way—i.e. in fighting—and their usual role, if any, has been a supporting one [4,9,17,29,31–35]. This strong sex difference is also observed in chimpanzees, which are our closest living relatives and are understood to be the only other primates that routinely engage in lethal intergroup conflict [11,36,37]. On the face of it, this pattern is puzzling because, if likelihood of success in warfare increases with the size of the war party, it is unclear why more than half of a group's potential warriors would almost always fail to participate in battle. The puzzle is not why male participation in warfare is more common than female participation (we outline potential explanations for this directly below), but why this imbalance is commonly so extreme, i.e. women taking no part at all.

Three general non-mutually exclusive hypotheses have been offered to explain this male bias in propensity to take part in warfare, and no general consensus has been reached. First, men might be predisposed to warfare because they are better at it. Specifically, having greater weight, height, and muscle mass may allow most men to perform more effectively in battle than most women [31]. Second, the net cost of warfare may be lower for men than women. In particular, while the fitness impact of risking death in battle may be significantly offset by a surviving warrior achieving great mating success, this is more likely to be true for men than for women owing to the way that male fitness can scale almost indefinitely with mating success, while female fitness has natural limits [9,17,31,38,39]. Also, the costs of participation in warfare are likely to be greater for women on account of the possibility of being pregnant or lactating, offspring survival being more strongly dependent on the continued presence of the mother than the father, sexual division of labour, and central place foraging leading to the opportunity costs of travelling being greater for women (e.g. [40]), and finally the risk of sexual coercion in case of defeat [9,31,41]. Third, women may be relatively less incentivized to participate in warfare owing to female-biased dispersal being associated with their having lower kinship to those group mates who stand to benefit in the event of success in warfare [36,38]. Female-biased dispersal (patrilocality) has been suggested to characterize ancestral humans [42] (but see [43]) and contemporary hunter–gatherers [44] (but see [45,46]), and is also observed in chimpanzees [47], in striking contradistinction to the usual mammalian syndrome of male-biased dispersal [48,49].

To formally assess the feasibility of these three hypotheses, and to explore how readily they explain extreme sex bias in participation in warfare—in terms of whether such sex differences are necessary and sufficient for exclusively male warfare to evolve—we extend an existing kin-selection model of exclusively male warfare [7,23] to incorporate participation by both men and women. We assume an infinite, group-structured population in which individuals disperse between groups with sex-specific probabilities, and then engage in warfare against other groups, with individual and group participation in warfare influencing the likelihood of enjoying reproductive success in one's own group and also in defeated groups (see Methods and electronic supplementary material for details). We use this model to investigate how natural selection might act to favour or disfavour male and/or female participation in warfare in the presence and absence of the previously hypothesized basic underlying sex differences. However, our key aim is to determine the conditions under which exclusively male participation in warfare might be expected to emerge as a stable evolutionary outcome.

## 2. Methods

We adapt and expand an existing model of exclusively male participation in warfare [7,23] so as to allow consideration of participation by both sexes. Specifically, we consider two coevolving traits: the tendency for a man to participate in war—i.e. the probability of joining the war party during either attack or defence—which we term male participation (*Ω*_m_), and the tendency for a woman to participate, which we term female participation (*Ω*_f_). Here, ‘participation’ is equivalent to the ‘bravery’ behaviour described in the exclusively male warfare versions of the model [7,23] (see electronic supplementary material for details). In the model, war is broadly construed so as to include, for example, surprise attacks as well as pitched battles. It is defined as an agonistic interaction between two groups, in which a subset of individuals of each group cooperate and coordinate to seize reproductive opportunities from the other group, as detailed below.

We consider an infinite population consisting of groups of *N*_i_ adults of sex 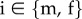. In the first step of the life cycle, each woman produces a large number *K*_i_ of sex-i offspring, who grow to become young adults (following [7,23], we assume non-overlapping generations, so that only young adult individuals—hereafter ‘individuals’—have the opportunity to migrate, fight and reproduce in each generation). Each sex-i individual migrates to a randomly chosen group with probability *m*_i_. In every generation, each post-migration group can attack one randomly chosen group, with probability *a*, and can be attacked by one other group, with the same probability *a*. If a war is initiated, a war party is formed in each of the two groups: each sex-i individual joins with probability *Ω*_i_. The attacking group wins with probability 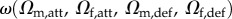, where *Ω*_i__,att_, and *Ω*_i__,def_ are the average probabilities of participation of sex-i individuals in the attacking and defending groups, respectively; and 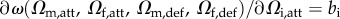 is the marginal increase in the probability of the attacking group winning, contributed by participation of sex 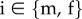 (we assume that participation has equal importance in defence: 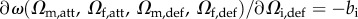). Density-dependent competition follows warfare. In groups that were not attacked, individuals compete for reproductive opportunities against group mates of the same sex; in groups that were attacked and successfully defended, individuals compete for reproductive opportunities against group mates of the same sex, with sex-i individuals having competitiveness *τ*_i_(*Ω*_i__,ind_)—where *Ω*_i__,ind_ is the probability of participation of a sex-i individual, and 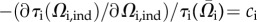 is the competitive cost of participation for an individual of sex i; and in defeated groups, individuals compete for reproductive opportunities against group mates and attackers of the same sex, with sex-i individuals having competitiveness 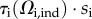 if they belong to the defeated group, and 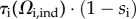 if they belong to the winning group. Notice that participation comes into play and incurs a competitive cost only when a group is involved in a war, either because it attacks or is attacked by another group. We perform a kin-selection analysis [50–57] to determine how selection acts upon male participation and female participation in warfare (see electronic supplementary material for details).

## 3. Results

Analysing our model, we find that natural selection—including both direct and indirect (i.e. kin selection) effects [50–57]—favours an increase in participation in warfare by an individual of sex i when
3.1
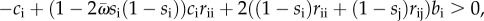
where *c*_i_ is the marginal cost of participation for that individual, *b*_i_ is the marginal increase in the probability of their group's victory, 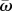 is the population-average probability of an attacking group being victorious, *s*_i_ is the proportion of children born into defeated groups whose sex-i parent was a member of the defeated—rather than a winning—group, *r*_ii_ is the genetic relatedness of same-sex group mates, and *r*_ij_ is the relatedness of opposite-sex group mates (both being lower than relatedness to self, which generates a collective action problem with tension between individual versus group interests). That is, by participating in warfare, an individual of sex i incurs: a direct-fitness cost (first term in condition (1)), owing to a loss −*c*_i_ of reproductive opportunities; an indirect-fitness benefit (second term), owing to a corresponding increase *c*_i_ of reproductive opportunities for other same-sex individuals, who are group mates with probability 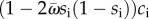 and in which case are related by *r*_ii_; and an indirect-fitness benefit (third term), from improving the group's success in warfare by *b*_i_ and consequently increasing the reproductive success of same-sex group mates—who are related by *r*_ii_—by a factor 1 − *s*_i_ and that of opposite-sex group mates—who are related by *r*_ij_—by a factor 1 − *s*_j_. Note that condition (1) holds even when individuals gain a direct fitness benefit from participating (*c*_i_ < 0; see electronic supplementary material for details), such as high prestige leading to increased mating success or other fitness-enhancing benefits [16,17], and may thus be satisfied even when group mates are not genetically related.

**Figure 1.**
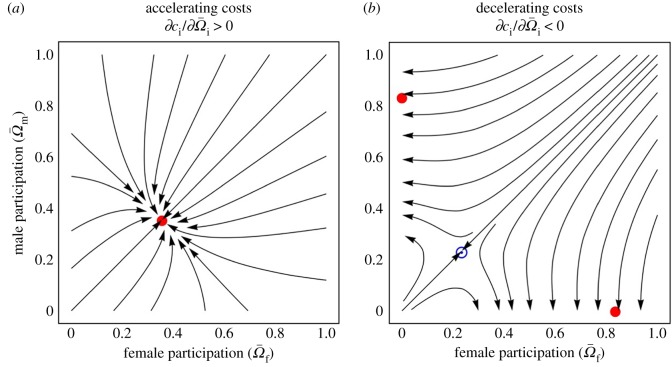
Evolution of male and female participation in the absence of other sex differences in the ecology of war. Streamline plots showing the evolution of male and female participation in warfare 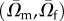 with accelerating personal costs (*a*) and with decelerating personal costs (*b*). Filled red circles represent stable equilibria and circled blue dots represent unstable equilibria. For the purposes of illustration, we assume competitiveness functional forms 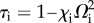 (*a*), and 
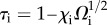
 (*b*), and a symmetrical war outcome function 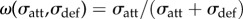, where 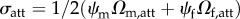 and 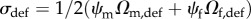 are the fighting strengths of the attacker and the defender, respectively (see electronic supplementary material for details), with *ψ*_f_ = *ψ*_m_ = 1 and *χ*_f_ = *χ*_m_ = 0.12. Other parameter values are 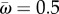, 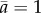, *N*_f_ = *N*_m_ = 10, *m*_m_ = *m*_f_ = 0.5, *s*_f_ = *s*_m_ = 0.15. (Online version in colour.)

To explore whether underlying sex differences are necessary for driving the evolution of single-sex participation, or whether this might occur for more basic reasons, we investigate the behaviour of our model in the simple, hypothetical case where these sex differences are absent. Consideration of condition (1) reveals that, even if there is no sex bias in any parameter and initially equal participation of both sexes in warfare (*c*_m_ = *c*_f_ = *c*, *b*_m_ = *b*_f_ = *b*, *m*_m_ = *m*_f_ = *m, s*_m_ = *s*_f_ = *s*), evolution may nevertheless result in single-sex participation in warfare, on account of a feedback that occurs within each sex. Specifically, the direct cost of participation in warfare manifests as a reduction in competitiveness against same-sex individuals for reproductive opportunities, and we find that if this marginal cost increases with increasing level of participation by members of one's own sex (‘accelerating cost’; 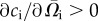, where 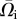 is the average level of participation in warfare by individuals of sex i), then the two sexes are favoured to participate equally (figure 1*a*), whereas if the marginal cost of participation decreases with increasing level of participation by members of one's own sex (‘decelerating cost’; 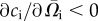), then single-sex participation is favoured (figure 1*b*). If constraints prevent a favoured increase in participation from one sex from being evolutionarily realized—for example, if that sex is already fully participating in warfare—then this may lead to the other sex also participating in warfare in compensation. Hence, if the cost of participation is decelerating, any initial symmetry-breaking sex bias in participation is expected to become evolutionarily magnified, such that whether the population evolves male-only or female-only participation depends only on the initial conditions (i.e. ‘hysteresis’).

This result reveals a fundamental role for sex in modulating selection pressures in relation to warfare such that—even in the absence of any other sex differences—the incentive for an individual to join a war party depends not only on how much other individuals are participating, but also on the individual's own sex and the sex of those other participating individuals. Specifically, an increased level of participation in warfare by sex-i individuals increases the incentive of a focal individual of the same sex to join the war party if
3.2
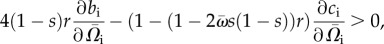
whereas it increases the incentive of a focal individual of the other sex to join the war party if
3.3
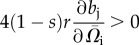
(see electronic supplementary material for details). In particular: if cost is accelerating 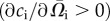, then the focal individual is relatively disincentivized to participate in warfare when same-sex individuals are already participating, leading to equal participation by both sexes being favoured (figure 1*a*); and if cost is decelerating 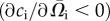, then the focal individual is relatively incentivized to participate in warfare when same-sex individuals are already participating, leading to only one sex being favoured to participate in warfare (figure 1*b*).

**Figure 2.**
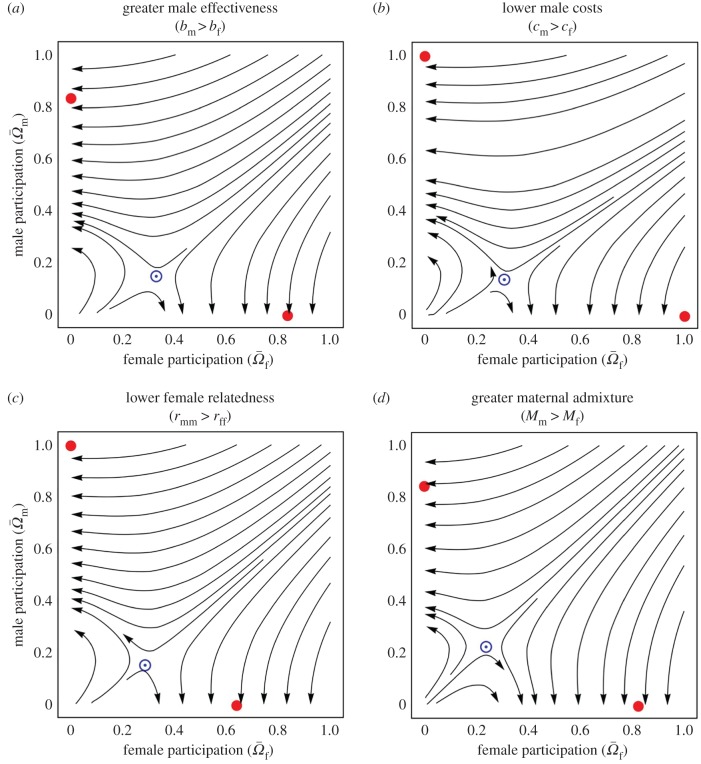
Evolution of male and female participation in the context of other sex differences in the ecology of war. Streamline plots showing the evolution of male and female participation in warfare 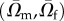 when personal costs are decelerating and: effectiveness is greater for men than for women (*b*_m_ > *b*_f_; *a*); men suffer lower personal costs than women (*c*_m_ < *c*_f_; *b*); women are less related to their same-sex group mates than men (*r*_mm_ > *r*_ff_; *c*) as a result of female-biased dispersal; maternal admixture is greater than paternal admixture (*M*_m_ < *M*_f_; *d*). Filled red circles represent stable equilibria and circled blue dots represent unstable equilibria. For the purposes of illustration, we assume a competitiveness functional form 
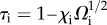
, and a symmetrical war outcome function 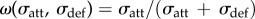, where 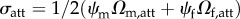 and 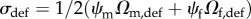 are the fighting strengths of the attacker and the defender, respectively (see electronic supplementary material for details). Other parameter values are 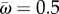, 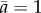, *N*_f_ = *N*_m_ = 10, *ψ*_f_ = *ψ*_m_ = 1 (except panel *a*: *ψ*_f_ = 0.7, *ψ*_m_ = 1), *χ*_f_ = *χ*_m_ = 0.12 (except panel *b*: *χ*_f_ = 0.14, *χ*_m_ = 0.1), *m*_f_ = *m*_m_ = 0.2 (except panel *c*: *m*_f_ = 0.3, *m*_m_ = 0.1), *s*_f_ = *s*_m_ = 0.15 (except panel *d*: *s*_f_ = 0.3, *s*_m_ = 0). (Online version in colour.)

These results explain why participation in warfare may involve one sex only, but not why participation in warfare is an exclusively male rather than exclusively female behaviour. To address this issue, we examine condition (1) to assess whether sex differences in various underlying parameters may bias this evolutionary exaggeration towards exclusively male participation rather than exclusively female participation. In support of the aforementioned hypotheses, we find that certain sex differences may result in a greater number of men than women participating in warfare (see electronic supplementary material for details). Moreover, we find that these sex differences may act in conjunction with the hysteresis effect described above to drive the evolution of exclusively male war parties, with no women participating. Specifically, the basin of attraction for male-only participation is larger than that for female-only participation if men are more effective than women in war (*b*_m_ > *b*_f_; figure 2*a*), if the cost of warfare is less for men than for women (*c*_m_ < *c*_f_; figure 2*b*), or if women disperse at a greater rate than do men (resulting in women being less related to same-sex group mates than men, *r*_ff_ < *r*_mm_; figure 2*c*). In each of these scenarios the left-hand side of condition (1) is larger for men than for women, tilting participation in their favour and making this outcome more likely (see electronic supplementary material for details). That is, starting from initially unbiased participation (for example, no participation by either sex), the population is expected to embark on an evolutionary trajectory that ultimately results in exclusively male warfare.

Moreover, we identify a further sex difference that may make exclusively male warfare more likely than exclusively female warfare. This obtains when the mothers of children born into a defeated group represent a mixture of women from winning and defeated groups (‘maternal admixture’ [23]) to an extent that is greater than for the fathers (‘paternal admixture’ [23]) of these children (*s*_f_(1 − *s*_f_) > *s*_m_(1 − *s*_m_)). This occurs, for example, when all men in defeated groups are killed and father no further children (*s*_m_ = 0) but some of the women are spared and go on to produce children (0 < *s*_f_ < 1). In such scenarios, a man who loses reproductive opportunities by participating in warfare is relatively more likely to reduce competition for reproductive opportunities among his male group mates, from which he may derive an indirect-fitness benefit, but a woman who loses reproductive opportunities by participating in warfare is relatively more likely to reduce competition among unrelated women in other groups. Accordingly, the left-hand side of condition (1) is larger for men than it is for women and, accordingly, the basin of attraction for male-only participation is larger than that for female-only participation (figure 2*d*). Again, this means that a population that initially exhibits unbiased participation is expected to evolve to a condition of exclusively male warfare.

## 4. Discussion

Our primary aim was to explore why human warfare has been not just a predominantly male activity but a near-exclusively male activity. To address this question, we developed and analysed a model of the co-evolution of male and female participation in warfare. Taken together, our results suggest an entirely novel explanation for why women do not participate in warfare. Archaeological, ethnographic, and historical evidence overwhelmingly show that warfare was an almost-exclusively male activity in prehistoric societies [24,26,27,29,30] and continued to be so in historical times in both small-scale societies and states throughout the ancient and modern world [4,9,17,29,31–35]. Although women fought occasionally in North-American and Melanesian tribes [34], in Scythian and Sarmatian steppe pastoralists—who may be linked to the Greek myth of the Amazons [31]—in the African Kingdom of Dahomey [58], and in Viking raiding parties [59], there is no evidence of war being a predominantly or exclusively female activity in any human society. We have shown that this pattern may be explained by an evolutionary feedback between male and female participation in warfare—specifically, increased participation of one sex incentivizing the same sex and disincentivizing the other—revealing that sex itself is a fundamental modulator of involvement in intergroup conflict. This effect ultimately owes to the way in which competition for Darwinian fitness is only between individuals of the same sex, and hence is not specific to our model but applies over a wide range of assumed societal organizations, generational differences, and migration patterns.

Considering only for illustration a simple hypothetical case with no sex difference in any underlying parameter (i.e. males and females are equally effective in war, pay equal costs of participation in war, gain equal direct benefits and indirect benefits to group mates, and migrate with equal rates), the evolution of male-only participation requires only two conditions be met: (i) that personal costs decelerate with increasing participation of individuals of the same sex; and (ii) that there is an initial symmetry-breaking male bias in participation. As an example of a scenario leading to condition (i) being satisfied, consider that a man who leaves the group to participate in warfare is less likely to be cuckolded by a group mate if his group mates are also participating in warfare. Let us now consider a potential scenario that would lead to condition (ii) being met. If warfare's origins lay in within-group aggression occasionally spilling out to the between-group level, then any pre-existing male bias in aggression—driven, for example, by standard sexual selection [60]—would have provided such an initial symmetry-breaking and thus ensure that subsequently evolving warfare behaviours were exclusively male in their expression. Thus, the empirically observed pattern of warfare being not just male-biased but, in most cases, an exclusively male activity can be explained under a very generally applicable set of circumstances.

Let us digress slightly to extend the line of reasoning related to warfare potentially having roots in within-group aggression. Warfare might therefore be conceptualized as a social innovation that allows wasteful sexually selected conflict among male group mates to be cooperatively redirected towards men in other groups, to the advantage of all group members. Beyond warfare, sexual feedbacks similar to those explored here may have played a role in the context of other group-beneficial social behaviours, such as communal care of infants and hunting, and might therefore explain striking patterns of sexual division of labour in ancestral—and to a certain extent contemporary—human societies [61,62].

Returning to our key aim of explaining why warfare is not just a predominantly male activity, but in most cases exclusively male: we have shown that, while they are not required for exclusively male warfare to evolve, any of a number of underlying sex differences may make this outcome more likely, by enlarging its basin of attraction, such that it encompasses initial conditions in which both sexes participate equally (including neither sex participating at all). Three such sex differences have previously been articulated in the literature. First, greater male than female effectiveness in warfare may result in a man having a greater positive impact on the probability of winning the war and a correspondingly greater increase in the reproductive success of his group mates than would a woman—in line with the rationale presented by Gat [30]. Second, lower costs for men than women make male-only participation more likely, as suggested by Low [17,38], Gat [31], and van Vugt [9]. Third, female-biased dispersal (patrilocality) increases the likelihood of male-only participation in two ways: it results in the indirect-fitness benefit accrued by men via increased breeding success for their group mates being greater for men than for women, as suggested by Manson & Wrangham [36] and Low [38]; and it also results in greater relaxation of kin competition for men than for women. As well as these three previously posited factors, our analysis suggests a further one that has previously been neglected: greater maternal than paternal admixture results in participation in warfare relaxing kin competition among men more than among women, thus making male-only participation more likely (for a similar effect driving sex-biased dispersal, see Micheletti *et al.* [23]).

Low [17,38] and Adams [32] have argued that while such underlying sex differences may drive a male bias in participation in warfare, they fail to convincingly explain why warfare is almost exclusively a male activity. Indeed, it was this criticism that was a spur for our investigations. Our analysis confirms this point: although each of these underlying factors may induce a quantitative male bias in participation in warfare, we find that the qualitative observation that participants in warfare are almost exclusively male is ultimately explained by the fundamentally sex-specific nature of Darwinian competition in sexual populations (men compete with men, and women with women). That is, although it has been suspected that underlying sex differences might not be sufficient to explain the evolution of exclusively male warfare, our analysis has shown that such sex differences are not even necessary.

The results of our model may also be applicable beyond humans. There is much controversy over the definition of warfare and, accordingly, as to which species should be regarded as exhibiting warfare behaviours [8,13,37]. However, the only vertebrates to have been observed to regularly engage in lethal conflict between conspecific groups are chimpanzees, spotted hyenas, wolves, and lions [37]. Our results offer a novel explanation for why, in chimpanzees, both attackers and victims are almost always male [11,33,37], and suggest that male philopatry—generally considered to be crucial in determining this pattern [36]—may simply be a reinforcing factor (along with other sex differences, such as in ranging patterns [63]). In spotted hyenas, only females participate in raids against other groups [37,64], and this suggests that the sexual feedbacks occurring in our model may apply in this case such that an initial female bias (e.g. in aggressiveness) might have led to female-only participation. Conversely, in wolves and lions, both sexes appear to take part in intergroup raids (though not necessarily in equal numbers [37,65,66]) which, in light of our analysis, suggests that personal costs might accelerate—rather than decelerate—with participation in these species. Finally, coalitionary killing is relatively common in many eusocial insects but, as their social systems (e.g. involving non-reproductive castes) and the modes and aims of their conflicts (e.g. attacking or defending against heterospecifics [67–70]) are fundamentally different from those considered in our analysis, it is not clear that our results would be applicable to those systems.

Returning to our own species, in addition to explaining the evolutionary origins of exclusively male warfare, our analysis may illuminate the biology of societally damaging violence-related pathologies in contemporary populations. Crespi & Badcock [71] have suggested that mutations and epimutations at loci controlling adaptive aggression behaviours may be linked with severe, psychotic-spectrum disorders, owing to the destabilizing effects of intragenomic conflict between an individual's maternal-origin versus paternal-origin genes, and Faria *et al*. [72] have pointed out that if adaptive aggression behaviours are sex-limited in their expression, then concomitant violence disorders are also expected to be sex-limited, perhaps explaining their higher incidence in men than in women. Crespi & Badcock [71] assumed that aggression is primarily a selfish, group-detrimental behaviour and, on that basis, predicted that psychotic-spectrum disorders are likely to be induced by deleterious mutations inherited from the individual's mother. However, if aggression has been primarily a selfless, group-beneficial behaviour—as in the case of participation in intergroup warfare—then the opposite pattern of parent-of-origin-specific expression is expected [23]. Moreover, the present analysis underlines why such pathologies may be male-biased in their incidence, i.e. owing to our species' almost-exclusively male participation in warfare.

Finally, our results may help illuminate the evolutionary trajectories of warfare as societies have changed and become more complex. Specifically, the presence of hysteresis—i.e. dependence on initial conditions and subsequent historical dynamics—might mean that, after an evolutionary equilibrium corresponding to a given set of initial biological and ecological conditions has been reached, the population is unlikely to move from that state, even if the conditions subsequently change. For example, in a society with male-only participation in warfare—which ancestrally had lower costs for men, female-biased migration and/or greater male effectiveness—almost-exclusively male involvement in warfare is likely to persist even if evolutionary innovations abolish sex differences in costs, effectiveness, and rates of migration. This might explain why war is almost exclusively the domain of men even in societies characterized by monogamy (in which there is less scope for men to enjoy limitless mating success) and in matrilocal, duolocal, and neolocal populations in which dispersal is not female-biased (e.g. Tibetan small-scale societies [73,74]). In addition, it suggests an explanation for why women did not participate more in warfare with the introduction of weapons that appear to decrease male advantage, such as the bow and arrow [18,31,32]. Similarly, the observation that in contemporary industrialized societies women's involvement in the armed forces is still considerably limited—though firepower and digitalization have, in many ways, equalized the sexes in terms of effectiveness in warfare [31]—need not be entirely due to cultural or ideological reasons, but might simply be a consequence of how ecological conditions faced by our ancestors have shaped our biology.

## Supplementary Material

Supporting_Information_ESM

## Data accessibility

This article has no additional data.
