# Supplementary material for: Why war is a man's game
Source: Proc Biol Sci. 2018 Aug 15;285(1884):20180975. doi: 10.1098/rspb.2018.0975 (PMC6111185; doi:10.1098/rspb.2018.0975)
Supplement: Supporting_Information_ESM [file rspb20180975supp1.pdf]

Supporting Information for

Why war is a man's game

Alberto J. C. Micheletti, Graeme D. Ruxton & Andy Gardner

Contents

1 Preamble ... 1

2 Fitness ... 2

3 Participation ... 3

3.1 Marginal fitness ... 3

3.2 Consanguinity and relatedness ... 4

4 Participation equilibria in the absence of other sex differences in the ecology of war ... 5

4.1 Feedbacks between male and female participation ... 10

5 Participation equilibria in the context of other sex differences in the ecology of war .. 11

5.1 Accelerating costs ... 11

5.2 Decelerating costs ... 12

6 Illustrations: war outcome functions ... 13

7 Additional references ... 14

1 Preamble

Here, we adapt an existing kin-selection model of exclusively-male warfare (7,23) to incorporate participation in warfare by both men and women. Specifically, we conceptualize the "bravery" trait – which in the studies by Lehmann & Feldman (7) and Micheletti et *al.* (23) measures the investment in warfare of a given individual – as the probability that that individual joins the war party when the group is involved in a war, and we term this trait "probability of participation" or "participation" in short. We consider the evolution of two traits: male participation, which is exhibited only by men, and female participation, which is exhibited only by women.

2 Fitness

Firstly, we derive the fitness of a focal young adult of sex i $\in$\{m,f\}, in a focal group, following the life cycle described in Methods. With probability $1 - m_i$, the focal young adult of sex-i does not migrate from the focal group. In every generation, each post-migration group can attack one randomly-chosen group. With probability $1 - a$, the focal group is not attacked by the other group and in this case the focal sex-i young adult competes for N_i breeding spots with $N_f K_i$ sex-i young adults (a fraction $1 - m_i$ of which originated from the focal group and a fraction m_i migrated from another group and in this way the migration terms cancel out; N_f is adult females) such that their probability of securing a breeding spot is $N_i/(N_f K_i)$. Alternatively, with probability a, the focal group is attacked and the attacking group loses the war with probability $1 - \omega''$, where $\omega'' \equiv \omega(\bar{\Omega}_m, \bar{\Omega}_f, \Omega_m', \Omega_f')$, $\bar{\Omega}_i$ is the average level of sex-i participation of the attacking group, and Ω_i' is the average level of sex-i participation of the focal group. In this case, the probability of obtaining reproductive success for a random sex-i young adult in the group must be corrected by $\tau_i(\Omega_i')$ and that of the focal sex-i young adult by $\tau_i(\Omega_i)$, where $\tau_i(\Omega_{i,ind})$ is sex-i competitiveness due to sex-i participation (with $d\tau_i/d\Omega_{i,ind} < 0$, and $\tau_i(0) = 1$), meaning that the focal sex-i young adult obtains a breeding spot with probability $N_i\tau_i(\Omega_i)/(N_f K_i\tau_i(\Omega_i'))$. Otherwise, the attacking group wins the war with probability ω''. In this case the focal sex-i young adult competes for $N_i s_i$ breeding spots with $N_f K_i s_i$ sex-i young adults from their group and $N_f K_i(1 - s_i)$ sex-i young adult from the attacking group. Thus, adding the appropriate competitiveness modifiers, the focal sex-i young adult obtains a breeding spot with probability $N_i\tau_i(\Omega_i)s_i/(N_f K_i\tau_i(\Omega_i')s_i + N_f K_i\tau_i(\bar{\Omega}_i)(1 - s_i))$. Further, the focal group may attack one other group. With probability $1 - a$, this does not happen, and in this case the focal sex-i young adult does not have the opportunity to obtain additional breeding spots. Alternatively, the focal group attacks the other group with probability a and loses the war with probability $1 - \omega'$, where $\omega' = \omega(\Omega_m', \Omega_f', \bar{\Omega}_m, \bar{\Omega}_f)$, which again results in no additional opportunities for breeding spots. Otherwise, the focal group wins with probability ω' and in this case the focal sex-i young adult has access to an additional $N_i(1 - s_i)$ breeding spots, for which he competes with $N_f K_i(1 - s_i)$ sex-i young adults from their group and $N_f K_i s_i$ sex-i young adults from the conquered group. Thus, adding the appropriate competitiveness modifiers, the focal sex-i young adult obtains a breeding spot in the defeated group with probability $N_i\tau_i(\Omega_i)(1 - s_i)/(N_f K_i\tau_i(\Omega_i')(1 - s_i) + N_f K_i\tau_i(\bar{\Omega}_i)s_i)$. Alternatively, the focal sex-i young adult migrates from the natal group with probability m_i to a randomly chosen group. In this case, fitness is identical to the philopatry case and the migration terms cancel out (N.B. migration rates influence the relatedness structure of the population, see *3.2. Consanguinity and relatedness*). Therefore, the absolute fitness of a focal sex-i young adult is equal to:

$$w_i = \left((1-a) + a\left((1-\omega'')\frac{\tau_i(\Omega_i)}{\tau_i(\Omega_i{}')} + \omega''\frac{\tau_i(\Omega_i)s_i}{\tau_i(\Omega_i{}')s_i + \tau_i(\bar{\Omega}_i)(1-s_i)}\right) + a\,\omega'\frac{\tau_i(\Omega_i)(1-s_i)}{\tau_i(\Omega_i{}')(1-s_i) + \tau_i(\bar{\Omega}_i)s_i}\right)\frac{N_i}{N_f K_i} \qquad \text{(A1)}$$

The average fitness of a sex-i young adult in the population is $\bar{w}_i = N_i/(N_f K_i)$ and the relative fitness of the focal sex-i young adult is given by $W_i = w_i / \bar{w}_i$.

3 Participation

3.1 Marginal fitness

In a sex-structured population, the relative fitness of an individual of unspecified sex is given by $W = c_m W_m + c_f W_f$ which is an average of the fitness of the two sexes, weighted by the class reproductive values of the two sexes, c_m and c_f (53,54,57,75,76). Consider a locus $\mathcal{G}$, which controls participation of sex i $\in$\{m,f\} Ω_i, a trait expressed only by young adults of sex i. Let g be the genic value of the focal individual for this gene, G the breeding value of the focal individual, G' the breeding value of a randomly-chosen groupmate of the focal individual, and $\bar{G}$ the average of the population. Under the assumption of vanishing genetic variation – all breeding values of the population clustered around the mean (53-54) – the direction of natural selection is given by:

$$\frac{\mathrm{d}W}{\mathrm{d}g} = c_m \frac{\mathrm{d}W_m}{\mathrm{d}g_m} + c_f \frac{\mathrm{d}W_f}{\mathrm{d}g_f}, \tag{A2}$$

with all derivatives evaluated at $g = g_m = g_f = \bar{G}$.

The derivative $\mathrm{d}W_i/\mathrm{d}g_i$ describes the impact of the genic value of a gene drawn from a young adult of sex i on their relative fitness. It can be expanded to reveal a direct fitness component (first addend), an indirect fitness component (second addend) and associations between genic values, breeding values and phenotypes (derivatives), obtaining:

$$\frac{\mathrm{d}W_i}{\mathrm{d}g_i} = \frac{\partial W_i}{\partial \Omega_i}\frac{\mathrm{d}\Omega_i}{\mathrm{d}G}\frac{\mathrm{d}G}{\mathrm{d}g_i} + \frac{\partial W_i}{\partial \Omega_i'}\frac{\mathrm{d}\Omega_i'}{\mathrm{d}G'}\frac{\mathrm{d}G'}{\mathrm{d}g_i} = \left(\frac{\partial W_i}{\partial \Omega_i} p_{self} + \frac{\partial W_i}{\partial \Omega_i'} p_{ii}\right)\gamma, \tag{A3}$$

where $\mathrm{d}W_i/\mathrm{d}\Omega_i$ is the impact of the participation phenotype of the focal sex-i individual on their fitness, $\mathrm{d}W_i/\mathrm{d}\Omega_i'$ is the impact of the participation phenotype of a random sex-i individual on the fitness of the focal sex-i individual, $p_{self} = \mathrm{d}G/\mathrm{d}g_i$ is the consanguinity of the focal individual of sex i to themselves, $p_{ii} = \mathrm{d}G'/\mathrm{d}g_i$ is the consanguinity between the focal individual of sex i and a random individual of the same sex in their group, and $\gamma = \mathrm{d}\Omega_i/\mathrm{d}G = \mathrm{d}\Omega_i'/\mathrm{d}G'$ is the correlation between an individual's phenotype and their breeding value.

Analogously, the derivative $\mathrm{d}W_j/\mathrm{d}g_j$ describes the impact of the genic value of a gene drawn from a young adult of the other sex on their relative fitness. It can be expanded to reveal the indirect fitness component (there is no direct fitness component, as the phenotype is expressed by young adults of sex i alone) and associations between genic values, breeding values and phenotypes (derivatives), obtaining:

$$\frac{\mathrm{d}W_j}{\mathrm{d}g_j} = \frac{\partial W_j}{\partial \Omega_i'}\frac{\mathrm{d}\Omega_i'}{\mathrm{d}G'}\frac{\mathrm{d}G'}{\mathrm{d}g_j} = \frac{\partial W_j}{\partial \Omega_i'} p_{ij}\,\gamma, \tag{A4}$$

where $dW_j/d\Omega_i'$ is the impact of the participation phenotype of a random sex-i individual on the fitness of the focal individual of the other sex, $p_{ij} = dG'/dg_j$ is the consanguinity between the focal individual of the other sex and a random sex-i individual in their group, and $\gamma = d\Omega_i'/dG'$ is the correlation between an individual's phenotype and their breeding value.

Both ω and τ_i are functions of Ω_i (see Methods). Therefore: $\partial W_i/\partial\Omega_i = (\partial W_i/\partial\tau_i(\Omega_i))(\partial\tau_i(\Omega_i)/\partial\Omega_i)$, $\partial W_i/\partial\Omega_i' = (\partial W_i/\partial\omega')(\partial\omega'/\partial\Omega_i') - (\partial W_i/\partial\omega'')(\partial\omega''/\partial\Omega_i') + (\partial W_i/\partial\tau_i(\Omega_i'))(\partial\tau_i(\Omega_i')/\partial\Omega_i')$, and $\partial W_j/\partial\Omega_i' = (\partial W_j/\partial\omega')(\partial\omega'/\partial\Omega_i') - (\partial W_j/\partial\omega'')(\partial\omega''/\partial\Omega_i')$, where $\partial\tau_i(\Omega_i)/\partial\Omega_i = \partial\tau_i(\Omega_i')/\partial\Omega_i' = -\tau_i(\bar{\Omega}_i)\, c_i$ and $\partial\omega'/\partial\Omega_i' = -\partial\omega''/\partial\Omega_i' = b_i$. Substituting these expressions and Eq. A3-4 into Eq. A2, we obtain:

$$\frac{dW}{dg} = c_i\left(\frac{\partial W_i}{\partial\tau_i(\Omega_i)}(-\tau_i(\bar{\Omega}_i)c_i)p_{self} + \left(\frac{\partial W_i}{\partial\omega'}b_i + \frac{\partial W_i}{\partial\omega''}b_i + \frac{\partial W_i}{\partial\tau_i(\Omega_i')}(-\tau_i(\bar{\Omega}_i)c_i)\right)p_{ii}\right)\gamma + c_j\left(\frac{\partial W_j}{\partial\omega'}b_i + \frac{\partial W_j}{\partial\omega''}b_i\right)p_{ij}\,\gamma \quad \text{(A5)}$$

Population average participation of sex i increases whenever the condition $dW/dg > 0$ is respected. Considering that $c_f = c_m = 1/2$ under diploid inheritance, this condition is given by:

$$-c_i p_{self} + \left(1 - 2\,\bar{\omega}\,s_i\,(1-s_i)\right)c_i p_{ii} + 2(1-s_i)b_i p_{ii} + 2\left(1-s_j\right)b_i p_{ij} > 0 \quad \text{(A6)}$$

Dividing by p_{self} to obtain $r_{ii} = p_{ii}/p_{self}$ and $r_{ij} = p_{ij}/p_{self}$ (see *3.2. Consanguinity and relatedness*) and rearranging terms yields condition [1] in the main text.

3.2 Consanguinity and relatedness

Given two individuals, A and B, and a locus, the coefficient of consanguinity between the two at that locus, p_{AB}, is equal to the probability of identity-by-descent between a gene randomly-drawn from that locus in individual A and a gene randomly-drawn from the same locus in individual B (77). In the case in which the second individual coincides with the first, i.e. B=A, the consanguinity of an individual to themselves is obtained and it is given by $p_{self} = (1 + f)/2$. The inbreeding coefficient f is the consanguinity between two mating partners, one from each sex, in a post-competition group and it is given by $f = \varphi_{mf}\, p_x$. Analogously, the consanguinity of two sex-i adults in a post-competition group is given by $p_{ii,adult} = \varphi_{ii}\, p_x$, where again i $\in$\{m,f\}. The coefficients $\varphi_{mf} = (1 - \bar{a}\,\bar{\omega}\,(2s_m s_f - s_m - s_f)(1 - m_m)(1 - m_f)$ and $\varphi_{ii} = (1 - 2\,\bar{a}\,\bar{\omega}\,s_i(1 - s_i))(1 - m_i)^2$ are the probabilities that two adults of opposite sex and two sex-i adults, respectively, who are in the same post-competition group, were born in the same group. p_x is the consanguinity of individuals born in the same group and is given by:

$$p_x = \frac{1}{4}\left(\frac{1}{N_m}p_{self} + \frac{N_m - 1}{N_m}p_{mm,adult}\right) + \frac{1}{2}f + \frac{1}{4}\left(\frac{1}{N_f}p_{self} + \frac{N_f - 1}{N_f}p_{ff,adult}\right) \quad \text{(A7)}$$

which, substituting the appropriate consanguinities and solving for p_x, becomes:

$$p_x = \frac{(N_m+N_f)\,\varphi_{mf}}{8\,N_m\,N_f - 2N_f(N_m-1)\,\varphi_{mm} - 2N_m(N_f-1)\,\varphi_{ff} - (4N_m\,N_f + N_m + N_f)\varphi_{mf}}. \quad \text{(A8)}$$

The consanguinity of a focal sex-i young adult to a random young adult of the same sex in their post-migration group is equal to the probability that they were born in the same group and that neither migrated, namely $p_{ii} = (1 - m_i)^2\, p_x$. Analogously, the consanguinity of the focal sex-i young adult to a random young adult of the opposite sex in their post-migration group is given by $p_{ij} = (1 - m_i)(1 - m_j)\, p_x$.

Given again two individuals, A and B, the coefficient of relatedness between the two, r_{AB}, is equal to the consanguinity of individual B to individual A, p_{AB}, divided by the consanguinity of individual B to themselves, $p_{BB} = p_{self}$ (77). Therefore, the relatedness of individuals born in the same group is given by $r_x = p_x/p_{self}$, the relatedness of a focal sex-i young adult to a random young adult of the same sex in their post-migration group is given by $r_{ii} = p_{ii}/p_{self} = (1 - m_i)^2\, r_x$ and the relatedness of a focal sex-i young adult to a random young adult of the other sex in their post-migration group is given by $r_{ij} = p_{ij}/p_{self} = (1 - m_i)(1 - m_j)\, r_x$.

4 Participation equilibria in the absence of other sex differences in the ecology of war

Consider a case in which there are no sex differences in the ecology of war other than potential differences in participation: $b_i(\Omega_i, \Omega_j) = b(\Omega_i, \Omega_j) = b(\Omega_j, \Omega_i)$, $c_i(\Omega_i) = c(\Omega_i)$, $m_i = m$, and $s_i = s$, for all i$\in${m,f}. It follows that $r_{ii} = r_{ij} = r$ and $s_i(1\text{-}s_i) = M_i = M$ (admixture). Therefore, the marginal fitness function for the participation of sex i is given by $\theta_i = 4(1-s)rb(\bar{\Omega}_i, \bar{\Omega}_j) - (1-(1-2\varpi M)r)c(\bar{\Omega}_i)$. Notice that the two marginal fitness functions for the two sexes are completely symmetrical. That is, one can be obtained from the other simply by exchanging i and j labels. This implies that, in a streamline plot (see Fig. 1), streamlines and equilibria for the participation of the two sexes are symmetrical with respect to the (0,0)-(1,1) diagonal, independently of the specific functional forms of c and b.

We now derive the condition for pairs of participation values of the two sexes to be equilibria and for these equilibria to be stable. For simplicity, we make three assumptions:

Assumption 1: The benefit function b is a strictly monotonically increasing or decreasing function of the participation of sex i. This can be expressed mathematically as:

$$\left(\frac{\partial b(\bar{\Omega}_i,\bar{\Omega}_j)}{\partial \bar{\Omega}_i}\Big|_{\bar{\Omega}_i=\hat{\Omega},\,\bar{\Omega}_j=\hat{\Omega}'} < 0\right) \vee \left(\frac{\partial b(\bar{\Omega}_i,\bar{\Omega}_j)}{\partial \bar{\Omega}_i}\Big|_{\bar{\Omega}_i=\hat{\Omega},\,\bar{\Omega}_j=\hat{\Omega}'} > 0\right) \forall \hat{\Omega}, \hat{\Omega}' \in [0,1]. \quad \text{(A9)}$$

Assumption 2: The cost function c is a strictly monotonically increasing or decreasing function of the participation of sex i. This can be expressed mathematically as:

$$\left(\frac{\partial c(\bar{\Omega}_i)}{\partial \bar{\Omega}_i}\Big|_{\bar{\Omega}_i=\hat{\Omega}} < 0\right) \vee \left(\frac{\partial c(\bar{\Omega}_i)}{\partial \bar{\Omega}_i}\Big|_{\bar{\Omega}_i=\hat{\Omega}} > 0\right) \forall \hat{\Omega} \in [0,1]. \quad \text{(A10)}$$

Assumption 3: The ratio of the benefit function and the cost function is a strictly monotonically increasing or decreasing function of the participation of sex i. This can be expressed mathematically as:

$$\left(\frac{\partial\left(\frac{b(\bar{\Omega}_{\mathrm{i}},\bar{\Omega}_{\mathrm{j}})}{c(\bar{\Omega}_{\mathrm{i}})}\right)}{\partial\bar{\Omega}_{\mathrm{i}}}\Big|_{\bar{\Omega}_i=\hat{\Omega},\,\bar{\Omega}_j=\hat{\Omega}'} < 0\right) \vee \left(\frac{\partial\left(\frac{b(\bar{\Omega}_{\mathrm{i}},\bar{\Omega}_{\mathrm{j}})}{c(\bar{\Omega}_{\mathrm{i}})}\right)}{\partial\bar{\Omega}_{\mathrm{i}}}\Big|_{\bar{\Omega}_i=\hat{\Omega},\,\bar{\Omega}_j=\hat{\Omega}'} > 0\right) \forall \hat{\Omega}, \hat{\Omega}' \in [0,1]. \quad \text{(A11)}$$

Considering that each participation term can assume either boundary (0 and 1) or intermediate values, we need to consider ten separate cases: the four corners of the square, the four sides, the diagonal, and the interior (non-diagonal).

i) $\bar{\Omega}_{\mathrm{i}} = 0$, for all i ∈{m,f}; The condition for this point to be a stable equilibrium is that the marginal fitness for participation of the two sexes are both negative, i.e. $\theta_{\mathrm{i}}|_{\bar{\Omega}_{\mathrm{i}}=0,\bar{\Omega}_{\mathrm{j}}=0} < 0$. If c(0)>0, this requires $b(0,0)/c(0) < P$, where $P = (1-(1-2\varpi M)r)/4(1-s)r$. If c(0)<0, this requires $b(0,0)/c(0) > P$, which can never be satisfied.

ii) $\bar{\Omega}_{\mathrm{i}} = 1$, for all i ∈{m,f}; The condition for this point to be a stable equilibrium is that the marginal fitness for participation of the two sexes are both positive, i.e. $\theta_{\mathrm{i}}|_{\bar{\Omega}_{\mathrm{i}}=1,\bar{\Omega}_{\mathrm{j}}=1} > 0$. If c(0)>0, this requires $b(1,1)/c(1) > P$. If c(0)<0, it requires $b(1,1)/c(1) < P$.

iii) $\bar{\Omega}_i = 1$ and $\bar{\Omega}_j = 0$, for all i ∈{m,f} and j ∈{m,f}, j≠i; The condition for this point to be a stable equilibrium is that the marginal fitness for participation of sex i is positive and that of the other sex is negative, i.e. $\theta_{\mathrm{i}}|_{\bar{\Omega}_{\mathrm{i}}=1,\bar{\Omega}_{\mathrm{j}}=0} > 0$ and $\theta_{\mathrm{j}}|_{\bar{\Omega}_{\mathrm{j}}=0,\bar{\Omega}_{\mathrm{i}}=1} < 0$. We consider the four possible cases:

1) If c(0)>0 and c(1)>0, the condition requires $b(0,1)/c(0) < P < b(1,0)/c(1)$, and considering Assumption 2, this implies $\partial c(\bar{\Omega}_{\mathrm{i}})/\partial\bar{\Omega}_{\mathrm{i}}|_{\bar{\Omega}_{\mathrm{i}}=\hat{\Omega}} < 0\ \forall\hat{\Omega} \in [0,1]$.
2) If c(0)<0 and c(1)>0, the condition requires $b(0,1)/c(0) > P$ and $b(1,0)/c(1) > P$, and the former is never satisfied.
3) If c(0)>0 and c(1)<0, the condition requires $b(1,0)/c(1) < b(0,1)/c(0) < P$, and considering Assumption 2, this implies $\partial c(\bar{\Omega}_{\mathrm{i}})/\partial\bar{\Omega}_{\mathrm{i}}|_{\bar{\Omega}_{\mathrm{i}}=\hat{\Omega}} < 0\ \forall\hat{\Omega} \in [0,1]$.
4) If c(0)<0 and c(1)<0, the condition requires $b(0,1)/c(0) > P$ and $b(1,0)/c(1) < P$, and the former is never satisfied.

iv) $\bar{\Omega}_i = 0$ and $\bar{\Omega}_j = 1$, for all i ∈{m,f} and j ∈{m,f}, j≠i; the condition for this point to be a stable equilibrium is identical to that of case iii). Therefore, when (1,0) is stable, (0,1) is also stable, and vice versa.

v) $\bar{\Omega}_{\mathrm{i}} = \tilde{\Omega} \neq 0{,}1$ and $\bar{\Omega}_{\mathrm{j}} = 0$, for all i ∈{m,f} and j ∈{m,f}, j≠i; The condition for this point to be an equilibrium is that the marginal fitness for participation of sex i is equal to zero and that of the other sex is negative, i.e. $\theta_{\mathrm{i}}|_{\bar{\Omega}_{\mathrm{i}}=\tilde{\Omega}\neq 0,1,\,\bar{\Omega}_{\mathrm{j}}=0} = 0$ and $\theta_{\mathrm{j}}|_{\bar{\Omega}_{\mathrm{j}}=0,\,\bar{\Omega}_{\mathrm{i}}=\tilde{\Omega}\neq 0,1} < 0$. We consider the four possible cases:

1) If $c(\tilde{\Omega}) > 0$ and $c(0)>0$, the condition requires $b(0,\tilde{\Omega})/c(0) < b(\tilde{\Omega},0)/c(\tilde{\Omega}) = P$. Considering Assumption 2, this implies $\partial c(\bar{\Omega}_{\mathrm{i}})/\partial \bar{\Omega}_{\mathrm{i}}|_{\bar{\Omega}_{\mathrm{i}}=\hat{\Omega}} < 0\ \forall \hat{\Omega} \in [0,1]$. If an equilibrium exists, the condition for it to be stable is that $\partial \theta_{\mathrm{i}}/\partial \bar{\Omega}_{\mathrm{i}}|_{\bar{\Omega}_{\mathrm{i}}=\tilde{\Omega}\neq 0,1,\,\bar{\Omega}_{\mathrm{j}}=0} < 0$, which requires $\partial b(\bar{\Omega}_{\mathrm{i}},\bar{\Omega}_{\mathrm{j}})/\partial \bar{\Omega}_{\mathrm{i}}|_{\bar{\Omega}_{\mathrm{i}}=\tilde{\Omega}\neq 0,1,\,\bar{\Omega}_{\mathrm{j}}=0} < P\partial b(\bar{\Omega}_{\mathrm{i}})/\partial \bar{\Omega}_{\mathrm{i}}|_{\bar{\Omega}_{\mathrm{i}}=\tilde{\Omega}\neq 0,1,\,\bar{\Omega}_{\mathrm{j}}=0}$. Considering Assumption 1, this implies $\left(\partial b(\bar{\Omega}_i,\bar{\Omega}_j)/\partial \bar{\Omega}_{\mathrm{i}}|_{\bar{\Omega}_i=\hat{\Omega},\,\bar{\Omega}_j=\hat{\Omega}'} < 0\right) \forall \hat{\Omega},\hat{\Omega}' \in [0,1]$.

2) If $c(\tilde{\Omega}) > 0$ and $c(0)<0$, the condition requires $b(\tilde{\Omega},0)/c(\tilde{\Omega}) = P$ and $b(0,\tilde{\Omega})/c(0) > P$, and the latter can never be satisfied.

3) If $c(\tilde{\Omega}) < 0$ and $c(0)>0$, the condition requires $b(\tilde{\Omega},0)/c(\tilde{\Omega}) = P$ and $b(0,\tilde{\Omega})/c(0) < P$, and the former can never be satisfied.

4)If $c(\tilde{\Omega}) < 0$ and $c(0)<0$, the condition requires $b(\tilde{\Omega},0)/c(\tilde{\Omega}) = P$ and $b(0,\tilde{\Omega})/c(0) > P$, and neither can be satisfied.

vi) $\bar{\Omega}_{\mathrm{i}} = 0$ and $\bar{\Omega}_{\mathrm{j}} = \tilde{\Omega} \neq 0{,}1$, for all i ∈{m,f} and j ∈{m,f}, j≠i; the condition for this point to be a stable equilibrium is identical to that of case v). Therefore, when$(\tilde{\Omega},0)$ is stable, $(0,\tilde{\Omega})$ is also stable, and vice versa.

vii) $\bar{\Omega}_{\mathrm{i}} = 1$ and $\bar{\Omega}_{\mathrm{j}} = \tilde{\Omega} \neq 0{,}1,$ for all $\mathrm{i} \in \{\mathrm{m},\mathrm{f}\}$ and $\mathrm{j} \in \{\mathrm{m},\mathrm{f}\}, \mathrm{j} \neq \mathrm{i}$; the condition for this point to be an equilibrium is that the marginal fitness for participation of sex i is positive and that of the other sex is equal to zero, i.e. $\theta_{\mathrm{i}}|_{\bar{\Omega}_{\mathrm{i}}=1,\,\bar{\Omega}_{\mathrm{j}}=\tilde{\Omega}\neq 0,1} > 0$ and $\theta_{\mathrm{j}}|_{\bar{\Omega}_{\mathrm{j}}=\tilde{\Omega}\neq 0,1\,,\,\bar{\Omega}_{\mathrm{i}}=1} = 0$. We consider the four possible cases:

1) If $c(\tilde{\Omega}) > 0$ and $c(1)>0$, the condition requires $b(1,\tilde{\Omega})/c(1) > b(\tilde{\Omega},1)/c(\tilde{\Omega}) = P$. Considering Assumption 2, this implies $\partial c(\bar{\Omega}_{\mathrm{i}})/\partial \bar{\Omega}_{\mathrm{i}}|_{\bar{\Omega}_{\mathrm{i}}=\hat{\Omega}} < 0\ \forall \hat{\Omega} \in [0,1]$. If an equilibrium exists, the condition for it to be stable is that $\partial \theta_{\mathrm{i}}/\partial \bar{\Omega}_{\mathrm{i}}|_{\bar{\Omega}_{\mathrm{i}}=1\,,\,\bar{\Omega}_{\mathrm{j}}=\tilde{\Omega}\neq 0,1} < 0$, which requires $\partial b(\bar{\Omega}_{\mathrm{i}},\bar{\Omega}_{\mathrm{j}})/\partial \bar{\Omega}_{\mathrm{j}}|_{\bar{\Omega}_{\mathrm{i}}=1,\,\bar{\Omega}_{\mathrm{j}}=\tilde{\Omega}\neq 0,1} < P\partial b(\bar{\Omega}_{\mathrm{j}})/\partial \bar{\Omega}_{\mathrm{j}}|_{\bar{\Omega}_{\mathrm{i}}=1,\,\bar{\Omega}_{\mathrm{j}}=\tilde{\Omega}\neq 0,1}$. Considering Assumption 1, this implies $\left(\partial b(\bar{\Omega}_i,\bar{\Omega}_j)/\partial \bar{\Omega}_{\mathrm{i}}|_{\bar{\Omega}_i=\hat{\Omega},\,\bar{\Omega}_j=\hat{\Omega}'} < 0\right) \forall \hat{\Omega},\hat{\Omega}' \in$ [0,1].

2) If $c(\tilde{\Omega}) > 0$ and $c(1)<0$, the condition which requires $b(1,\tilde{\Omega})/c(1) < b(\tilde{\Omega},1)/c(\tilde{\Omega}) = P$. Considering Assumption 2, this implies $\partial c(\bar{\Omega}_{\mathrm{i}})/\partial \bar{\Omega}_{\mathrm{i}}|_{\bar{\Omega}_{\mathrm{i}}=\hat{\Omega}} < 0\ \forall \hat{\Omega} \in [0,1]$. If an equilibrium exists, the condition for it to be stable is that $\partial \theta_{\mathrm{i}}/\partial \bar{\Omega}_{\mathrm{i}}|_{\bar{\Omega}_{\mathrm{i}}=1\,,\,\bar{\Omega}_{\mathrm{j}}=\tilde{\Omega}\neq 0,1} < 0$, which requires $\partial b(\bar{\Omega}_{\mathrm{i}},\bar{\Omega}_{\mathrm{j}})/\partial \bar{\Omega}_{\mathrm{j}}|_{\bar{\Omega}_{\mathrm{i}}=1,\,\bar{\Omega}_{\mathrm{j}}=\tilde{\Omega}\neq 0,1} < P\partial b(\bar{\Omega}_{\mathrm{j}})/\partial \bar{\Omega}_{\mathrm{j}}|_{\bar{\Omega}_{\mathrm{i}}=1,\,\bar{\Omega}_{\mathrm{j}}=\tilde{\Omega}\neq 0,1}$.

Considering Assumption 1, this implies $\left(\partial b(\bar{\Omega}_i, \bar{\Omega}_j)/\partial\bar{\Omega}_\mathrm{i}|_{\bar{\Omega}_i=\hat{\Omega}, \bar{\Omega}_j=\hat{\Omega}'} < 0\right) \forall \hat{\Omega}, \hat{\Omega}' \in$ [0,1].

3) If $c(\tilde{\Omega}) < 0$ and c(1)>0, the condition requires $b(\tilde{\Omega}, 1)/c(\tilde{\Omega}) = P$ and $b(1, \tilde{\Omega})/c(1) > P$, and the former can never be satisfied.

4)If $c(\tilde{\Omega}) < 0$ and c(1)<0, the condition requires $b(\tilde{\Omega}, 1)/c(\tilde{\Omega}) = P$ and $\frac{b(1,\tilde{\Omega})}{c(1)} < P$, and the former can never be satisfied.

viii) $\bar{\Omega}_\mathrm{i} = \tilde{\Omega} \neq 0{,}1$ and $\bar{\Omega}_\mathrm{j} = 1$, for all i ∈{m,f} and j ∈{m,f}, j≠i; the condition for this point to be a stable equilibrium is identical to that of case vii). Therefore, when$(\tilde{\Omega}, 1)$ is stable, $(1, \tilde{\Omega})$ is also stable, and vice versa.

ix) $\bar{\Omega}_\mathrm{i} = \tilde{\Omega} \neq 0{,}1$, for all i∈{m,f}; The condition for a point on the diagonal to be an equilibrium is that the marginal fitness for participation of sex i is equal to that of the other sex and both are equal to zero, i.e. $\theta_\mathrm{i}|_{\bar{\Omega}_\mathrm{i}=\tilde{\Omega}\neq 0,1} = 0$, which requires $b(\bar{\Omega}_\mathrm{i}, \bar{\Omega}_\mathrm{j})/c(\bar{\Omega}_\mathrm{i})|_{\bar{\Omega}_\mathrm{i}=\tilde{\Omega}\neq 0,1} = P$. This condition can only be satisfied if $c(\bar{\Omega}_\mathrm{i})|_{\bar{\Omega}_\mathrm{i}=\tilde{\Omega}\neq 0,1} > 0$. Considering Assumption 3, there cannot be more than one equilibrium on the diagonal. If an equilibrium exists, the condition for it to be stable is that both eigenvalues of the Jacobian matrix of the model are negative. The Jacobian matrix of the model is:

$$J = \begin{pmatrix} \frac{\partial\theta_\mathrm{i}}{\partial\bar{\Omega}_\mathrm{i}} & \frac{\partial\theta_\mathrm{i}}{\partial\bar{\Omega}_\mathrm{j}} \\ \frac{\partial\theta_\mathrm{i}}{\partial\bar{\Omega}_\mathrm{j}} & \frac{\partial\theta_\mathrm{i}}{\partial\bar{\Omega}_\mathrm{i}} \end{pmatrix} \qquad \text{(A12)}$$

The condition for both eigenvalues of the matrix to be negative is $\partial c(\bar{\Omega}_\mathrm{i})/\partial\bar{\Omega}_\mathrm{i}|_{\bar{\Omega}_\mathrm{i}=\hat{\Omega}} > 0 \ \forall \hat{\Omega} \in$ [0,1] and $\partial b(\bar{\Omega}_\mathrm{i}, \bar{\Omega}_\mathrm{j})/\partial\bar{\Omega}_\mathrm{i}|_{\bar{\Omega}_\mathrm{i}=\tilde{\Omega}\neq 0,1} = ½P\partial c(\bar{\Omega}_\mathrm{i})/\partial\bar{\Omega}_\mathrm{i}|_{\bar{\Omega}_\mathrm{i}=\tilde{\Omega}\neq 0,1}$.

x) $\bar{\Omega}_\mathrm{i} \neq \bar{\Omega}_\mathrm{j} \neq 0{,}1$, for all i ∈{m,f} and j ∈{m,f}, j≠i; the marginal fitness functions for participation of the two sexes are identical with the exception of $c(\bar{\Omega}_\mathrm{i}) = c(\bar{\Omega}_\mathrm{j})$. The condition for this point to be a stable equilibrium is that $\theta_\mathrm{i}|_{\bar{\Omega}_\mathrm{i}\neq\bar{\Omega}_\mathrm{j}\neq 0,1} = \theta_\mathrm{j}|_{\bar{\Omega}_\mathrm{i}\neq\bar{\Omega}_\mathrm{j}\neq 0,1} = 0$, which requires $b(\bar{\Omega}_\mathrm{i}, \bar{\Omega}_\mathrm{j})/c(\bar{\Omega}_\mathrm{i})|_{\bar{\Omega}_\mathrm{i}\neq\bar{\Omega}_\mathrm{j}\neq 0,1} = b(\bar{\Omega}_\mathrm{j}, \bar{\Omega}_\mathrm{i})/c(\bar{\Omega}_\mathrm{j})|_{\bar{\Omega}_\mathrm{i}\neq\bar{\Omega}_\mathrm{j}\neq 0,1} = P$. Since $\bar{\Omega}_\mathrm{i} \neq \bar{\Omega}_\mathrm{j}$, this is impossible. Points $(\bar{\Omega}_\mathrm{i}, \bar{\Omega}_\mathrm{j})$ with $\bar{\Omega}_\mathrm{i} \neq \bar{\Omega}_\mathrm{j} \neq 0{,}1$ can never be equilibria.

We now seek to demonstrate that nine equilibria configurations are not allowed. We consider them in turn:

I) stable equilibria at $(\tilde{\Omega}, 0)$ with $\tilde{\Omega} \neq 0{,}1$ and (1,0); this requires $b(\tilde{\Omega}, 0)/c(\tilde{\Omega}) < b(1{,}0)/c(1)$. Since costs are required to be decelerating, it follows that $c\big(\tilde{\Omega}\big) > c(1)$. As a consequence, $b(1{,}0) > b(\tilde{\Omega}, 0)$, but this is impossible because benefits are required to be decelerating. This stable equilibria configuration is thus not allowed.

II) stable equilibria at $(0, \tilde{\Omega})$ with $\tilde{\Omega} \neq 0{,}1$ and $(0{,}1)$; this case is analogous to case I) above.

III) stable equilibria at $(1, \tilde{\Omega})$ with $\tilde{\Omega} \neq 0{,}1$ and (1,0) this requires $b(\tilde{\Omega}, 1)/b(1{,}0) < c(\tilde{\Omega})/c(1)$. Since costs are required to be decelerating, it follows that $c\big(\tilde{\Omega}\big) > c(1)$. As a consequence, $b(1{,}0) > b(\tilde{\Omega}, 0)$, but this is impossible because benefits are required to be decelerating. This stable equilibria configuration is thus not allowed.

IV) stable equilibria at $(\tilde{\Omega}, 1)$ with $\tilde{\Omega} \neq 0{,}1$ and $(0{,}1)$; this case is analogous to case III) above.

V) stable equilibria at $(\tilde{\Omega}, \tilde{\Omega})$ with $\tilde{\Omega} \neq 0{,}1$ and $(0, 0)$; this requires $b(0{,}0)/c(0) < b(1{,}1)/c(1)$, which itself requires $\partial(b(\bar{\Omega}_\mathrm{i}, \bar{\Omega}_\mathrm{j})/c(\bar{\Omega}_\mathrm{i}))/\partial\bar{\Omega}_\mathrm{i} > 0$ and as a consequence $(\partial b(\bar{\Omega}_\mathrm{i}, \bar{\Omega}_\mathrm{j})/ \partial\bar{\Omega}_\mathrm{i})c(\bar{\Omega}_\mathrm{i}) - b(\bar{\Omega}_\mathrm{i}, \bar{\Omega}_\mathrm{j})(\partial c(\bar{\Omega}_\mathrm{i})/\partial\bar{\Omega}_\mathrm{i}) > 0$ which contradicts part of the condition for the first point to be an equilibrium (i.e. $\partial b(\bar{\Omega}_\mathrm{i}, \bar{\Omega}_\mathrm{j})/\partial\bar{\Omega}_\mathrm{i}|_{\bar{\Omega}_\mathrm{i}=\tilde{\Omega} \neq 0,1} = ½P\partial c(\bar{\Omega}_\mathrm{i})/\partial\bar{\Omega}_\mathrm{i}|_{\bar{\Omega}_\mathrm{i} =\tilde{\Omega}\neq 0,1}$, see case ix) above).

VI) stable equilibria at $(\tilde{\Omega}, \tilde{\Omega})$ with $\tilde{\Omega} \neq 0{,}1$ and $(0, 0)$; this case is analogous to case V) above.

VII) stable equilibria at $(\tilde{\Omega}, \tilde{\Omega})$ with $\tilde{\Omega} \neq 0{,}1$ and $(1, 0)$ (or $(1, 0)$); the first point requires accelerating costs, while the second one requires decelerating costs. This stable equilibria configuration is thus not allowed.

VIII) stable equilibria at $(\tilde{\Omega}, \tilde{\Omega})$ with $\tilde{\Omega} \neq 0{,}1$ $(0, \tilde{\Omega}')$ with $\tilde{\Omega}' \neq 0{,}1$ (or $(\tilde{\Omega}', 0)$); the first point requires accelerating costs, while the second one requires decelerating costs. This stable equilibria configuration is thus not allowed.

IX) stable equilibria at $(\tilde{\Omega}, \tilde{\Omega})$ with $\tilde{\Omega} \neq 0{,}1$ $(1, \tilde{\Omega}')$ with $\tilde{\Omega}' \neq 0{,}1$ (or $(\tilde{\Omega}', 1)$); the first point requires accelerating costs, while the second one requires decelerating costs. This stable equilibria configuration is thus not allowed.

Therefore, for any given set of parameters, two classes of stable equilibria configurations may be obtained depending on the cost function:

a) If costs are accelerating ($\partial c(\bar{\Omega}_\mathrm{i})/\partial\bar{\Omega}_\mathrm{i}|_{\bar{\Omega}_\mathrm{i}=\hat{\Omega}} > 0\ \forall\hat{\Omega} \in [0,1]$), there is always at least one stable equilibrium, either: a.1) one and only one stable equilibrium on the diagonal ($\tilde{\Omega}, \tilde{\Omega}$), extremes included; or a.2) a stable equilibrium on (0,0) and one on (1,1).

b) If costs are decelerating ($\partial c(\bar{\Omega}_\mathrm{i})/\partial\bar{\Omega}_\mathrm{i}|_{\bar{\Omega}_\mathrm{i}=\hat{\Omega}} > 0\ \forall\hat{\Omega} \in [0,1]$), there is always at least one stable equilibrium, which cannot be on the diagonal. All equilibria configurations not excluded in cases I-IX) above are allowed and therefore possible configurations are: b.1) (0,0) and (1,1); b.2) (0,0); b.3) (1,1); b.4) ($0, \tilde{\Omega}$) and ($\tilde{\Omega}, 0$); b.5) ($1, \tilde{\Omega}$) and ($\tilde{\Omega}, 1$); b.6) ($0, \tilde{\Omega}$), ($\tilde{\Omega}, 0$), ($1, \tilde{\Omega}'$) and ($\tilde{\Omega}', 1$); b.7) (0,1) and (1,0); b.8) (0,1), (1,0), (0,0); b.9) (0,1), (1,0), (1,1).

4.1 Feedbacks between male and female participation

The impact of increased participation of young adults (hereafter, "individuals") of sex i ∈{m,f} on participation of individuals of the same sex is given by:

$$\frac{\partial\theta_\mathrm{i}}{\partial\bar{\Omega}_\mathrm{i}} = 4(1-s)r\frac{\partial b_\mathrm{i}}{\partial\bar{\Omega}_\mathrm{i}} - \big(1-\big(1-2\bar{\omega}s(1-s)\big)r\big)\frac{\partial c_\mathrm{i}}{\partial\bar{\Omega}_\mathrm{i}}, \qquad \text{(A13)}$$

which is equal to the l.h.s. of condition [2] in the main text. Analogously, the impact of increased participation of individuals of sex i on participation of individuals of the other sex is given by:

$$\frac{\partial\theta_\mathrm{j}}{\partial\bar{\Omega}_\mathrm{i}} = 4(1-s)r\frac{\partial b_\mathrm{j}}{\partial\bar{\Omega}_\mathrm{i}}, \qquad \text{(A14)}$$

which is equal to the l.h.s. of condition [3] in the main text.

Whether personal costs for individuals of one sex increase with increasing participation of that same sex ("accelerating costs") or decrease with increasing participation of that same sex ("decelerating costs"), determines the nature of the feedbacks between male and female participation. Personal costs of warfare consist in a loss of competitive ability for breeding spots. Competition for reproduction is fundamentally within-sex, because the total reproductive value of each sex is fixed (and, indeed, in this non-overlapping generations scenario, they are both equal to one half (75,78)).

If costs are accelerating ($\partial c(\bar{\Omega}_\mathrm{i})/\partial\bar{\Omega}_\mathrm{i}|_{\bar{\Omega}_\mathrm{i}=\hat{\Omega}} > 0\ \forall\hat{\Omega} \in [0,1]$), an increase in participation of sex-i individuals results in other sex-i individuals being relatively disincentivized to participate in warfare and individuals of the other sex being relatively incentivized to participate in warfare, that is $\partial\theta_\mathrm{i}/\partial\bar{\Omega}_\mathrm{i} < \partial\theta_\mathrm{j}/\partial\bar{\Omega}_\mathrm{i}$ (see also Fig. 1a). Specifically, three cases are possible: if $\partial\theta_\mathrm{i}/\partial\bar{\Omega}_\mathrm{i} > 0$ and $\partial\theta_\mathrm{j}/\partial\bar{\Omega}_\mathrm{i} > 0$, an increase in participation of sex-i individuals promotes further participation of individuals of the same sex less than participation of individuals of the other sex; if $\partial\theta_\mathrm{i}/\partial\bar{\Omega}_\mathrm{i} < 0$ and $\partial\theta_\mathrm{j}/\partial\bar{\Omega}_\mathrm{i} > 0$, an increase in participation of sex-i individuals inhibits further participation of individuals of the same sex and promotes participation of individuals of the other

sex; if $\partial\theta_i/\partial\bar{\Omega}_i < 0$ and $\partial\theta_j/\partial\bar{\Omega}_i < 0$, an increase in participation of sex-i individuals inhibits further participation of individuals of the same sex more than participation of individuals of the other sex.

Analogously, if costs are decelerating ($\partial c(\bar{\Omega}_i)/\partial\bar{\Omega}_i|_{\bar{\Omega}_i=\hat{\Omega}} < 0\ \forall\hat{\Omega} \in [0,1]$), an increase in participation of sex-i individuals results in other sex-i individuals being relatively incentivized to participate in warfare and individuals of the other sex being relatively disincentivized to participate in warfare, that is $\partial\theta_i/\partial\bar{\Omega}_i > \partial\theta_j/\partial\bar{\Omega}_i$ (see also Fig. 1b). Specifically, three cases are possible: if $\partial\theta_i/\partial\bar{\Omega}_i > 0$ and $\partial\theta_j/\partial\bar{\Omega}_i > 0$, an increase in participation of sex-i individuals promotes further participation of individuals of the same sex more than participation of individuals of the other sex; if $\partial\theta_i/\partial\bar{\Omega}_i > 0$ and $\partial\theta_j/\partial\bar{\Omega}_i < 0$, an increase in participation of sex-i individuals promotes further participation of individuals of the same sex and inhibits participation of individuals of the other sex; if $\partial\theta_i/\partial\bar{\Omega}_i < 0$ and $\partial\theta_j/\partial\bar{\Omega}_i < 0$, an increase in participation of sex-i individuals inhibits further participation of individuals of the same sex less than participation of individuals of the other sex.

5 Participation equilibria in the context of other sex differences in the ecology of war

Consider now a case in which there may be sex differences in the ecology of warfare other than differences in participation.

5.1 Accelerating costs

We demonstrate that, in the case in which personal costs are accelerating, and the benefits, or personal costs, or migration rates, or admixture coefficients are not equal for the two sexes, a population with equal participation of the two sexes – i.e. a point on the diagonal, excluding the extremes – cannot be in a stable equilibrium, and therefore it will be pushed off of the diagonal. In addition, a difference in in one of the four ecological parameters listed above is sufficient condition for $\Omega_i^* \geq \Omega_j^*$ or $\Omega_i^* \leq \Omega_j^*$, where Ω_i^* is the stable level of participation for sex i $\in$\{m,f\}. We consider four cases in turn:

i) $b_i(\Omega_i, \Omega_j) \neq b_j(\Omega_i, \Omega_j)$, and $c_i(\Omega_i) = c(\Omega_i)$, $m_i = m$, $s_i = s$, which implies $M_i = M$, for all i $\in$\{m,f\}; considering a population characterized by $(\tilde{\Omega}, \tilde{\Omega})$ with $\tilde{\Omega} \neq 0,1$, the marginal fitness function for participation of sex i is $\theta_i = 4(1-s)rb_i(\tilde{\Omega}, \tilde{\Omega}) - (1-(1-2\varpi M)r)c(\tilde{\Omega})$. If $b_i > b_j$, then $\theta_i > \theta_j$ for every possible value of $\tilde{\Omega}$, which implies $\Omega_i^* \geq \Omega_j^*$.

ii) $c_i(\Omega_i) \neq c_j(\Omega_i)$ and $b_i(\Omega_i, \Omega_j) = b(\Omega_i, \Omega_j) = b(\Omega_j, \Omega_i)$, $m_i = m$, $s_i = s$, which implies $M_i = M$, for all i $\in$\{m,f\}; considering a population characterized by $(\tilde{\Omega}, \tilde{\Omega})$ with $\tilde{\Omega} \neq 0,1$, the marginal fitness function for participation of sex i is $\theta_i = 4(1-s)rb(\tilde{\Omega}, \tilde{\Omega}) - (1-(1-2\varpi M)r)c_i(\tilde{\Omega})$. If $c_i < c_j$, then $\theta_i > \theta_j$ for every possible value of $\tilde{\Omega}$, which implies $\Omega_i^* \geq \Omega_j^*$.

iii) $m_i \neq m_j$ and $b_i(\Omega_i, \Omega_j) = b(\Omega_i, \Omega_j) = b(\Omega_j, \Omega_i)$, $c_i(\Omega_i) = c(\Omega_i)$, $s_i = s$, which implies $M_i = M$, for all $i \in \{m,f\}$; considering a population characterized by $(\tilde{\Omega}, \tilde{\Omega})$ with $\tilde{\Omega} \neq 0,1$, the marginal fitness function for participation of sex i is $\theta_i = 2\left((1-s)(r_{ii}+r_{ij})\right)b(\tilde{\Omega}, \tilde{\Omega}) - (1-(1-2\varpi M)r_{ii})c_i(\tilde{\Omega})$, where $r_{ii} = (1-m_i)^2 r_x$ and $r_{ij} = (1-m_i)(1-m_j)r_x$. If $m_i < m_j$, then $\theta_i > \theta_j$ for every possible value of $\tilde{\Omega}$, which implies $\Omega_i^* \geq \Omega_j^*$.

iv) $M_i \neq M_j$ and $b_i(\Omega_i, \Omega_j) = b(\Omega_i, \Omega_j) = b(\Omega_j, \Omega_i)$, $c_i(\Omega_i) = c(\Omega_i)$, $m_i = m$, for all $i \in \{m,f\}$; considering a point on the diagonal $(\tilde{\Omega}, \tilde{\Omega})$ with $\tilde{\Omega} \neq 0,1$, the marginal fitness function for participation of sex i is $\theta_i = 2\left((1-s_i)+(1-s_j)\right)rb(\tilde{\Omega}, \tilde{\Omega}) - (1-(1-2\varpi M_i)r)c(\tilde{\Omega})$. If $M_i < M_j$, then $\theta_i > \theta_j$ for every possible value of $\tilde{\Omega}$, which implies $\Omega_i^* \geq \Omega_j^*$.

5.2 Decelerating costs

We demonstrate that, in the case in which personal costs are decelerating, and the benefits, or personal costs, or migration rates, or admixture coefficients are not equal for the two sexes, a population with equal participation of the two sexes – i.e. a point on the diagonal, excluding the extremes – cannot be a stable equilibrium, and therefore it will be pushed off of the diagonal. This implies that, in the case in which two single-sex equilibria exist, the diagonal is contained in one of the two subspaces of initial conditions leading to such equilibria and therefore the two subspaces (basins of attraction) are of unequal size. We consider four cases in turn:

i) $b_i(\Omega_i, \Omega_j) \neq b_j(\Omega_i, \Omega_j)$, and $c_i(\Omega_i) = c(\Omega_i)$, $m_i = m$, $s_i = s$, which implies $M_i = M$, for all $i \in \{m,f\}$; considering a population characterized by $(\tilde{\Omega}, \tilde{\Omega})$ with $\tilde{\Omega} \neq 0,1$, the marginal fitness function for participation of sex i is $\theta_i = 4(1-s)rb_i(\tilde{\Omega}, \tilde{\Omega}) - (1-(1-2\varpi M)r)c(\tilde{\Omega})$. If $b_i > b_j$, then $\theta_i > \theta_j$ for every possible value of $\tilde{\Omega}$, and in this case selection will drive the population off of the diagonal, and specifically towards the sex-i-only equilibrium, which implies that the basin of attraction for the sex-i-only equilibrium encompasses the diagonal and is therefore larger than the basin of attraction for the equilibrium where only the other sex participates.

ii) $c_i(\Omega_i) \neq c_j(\Omega_i)$ and $b_i(\Omega_i, \Omega_j) = b(\Omega_i, \Omega_j) = b(\Omega_j, \Omega_i)$, $m_i = m$, $s_i = s$, which implies $M_i = M$, for all $i \in \{m,f\}$; considering a population characterized by $(\tilde{\Omega}, \tilde{\Omega})$ with $\tilde{\Omega} \neq 0,1$, the marginal fitness function for participation of sex i is $\theta_i = 4(1-s)rb(\tilde{\Omega}, \tilde{\Omega}) - (1-(1-2\varpi M)r)c_i(\tilde{\Omega})$. If $c_i < c_j$, then $\theta_i > \theta_j$ for every possible value of $\tilde{\Omega}$, and in this case selection will drive the population off of the diagonal, and specifically towards the sex-i-only equilibrium, which implies that the basin of attraction for the sex-i-only equilibrium encompasses the diagonal and is therefore larger than the basin of attraction for the equilibrium where only the other sex participates. N.B. $c_i < c_j$ also results in a decrease in the indirect-fitness benefit sex-i individuals accrue via lost

reproductive opportunities in their home group being taken by their groupmates of the same sex (relaxation of kin competition; see second term in condition [1]). However, this benefit never outweighs the direct inclusive fitness cost associated with a loss of competitiveness (first term in condition [1]) and therefore lower personal costs for sex-*i* individuals always lead to the sex-i-only outcome being more likely.

iii) $m_i \neq m_j$ and $b_i(\Omega_i, \Omega_j) = b(\Omega_i, \Omega_j) = b(\Omega_j, \Omega_i)$, $c_i(\Omega_i) = c(\Omega_i)$, $s_i = s$, which implies $M_i = M$, for all $i \in\{m,f\}$; considering a population characterized by $(\tilde{\Omega},\tilde{\Omega})$ with $\tilde{\Omega} \neq 0,1$, the marginal fitness function for participation of sex i is $\theta_i = 2\Big((1-s)(r_{ii}+r_{ij})\Big) b(\tilde{\Omega},\tilde{\Omega}) - (1-(1-2\varpi M) r_{ii}) c_i\big(\tilde{\Omega}\big)$, where $r_{ii} = (1-m_i)^2 r_x$ and $r_{ij} = (1-m_i)\big(1-m_j\big) r_x$. If $m_i < m_j$, then $\theta_i > \theta_j$ for every possible value of $\tilde{\Omega}$, and in this case selection will drive the population off of the diagonal, and specifically towards the sex-i-only equilibrium, which implies that the basin of attraction for the sex-i-only equilibrium encompasses the diagonal and is therefore larger than the basin of attraction for the equilibrium where only the other sex participates.

iv) $M_i \neq M_j$ and $b_i(\Omega_i, \Omega_j) = b(\Omega_i, \Omega_j) = b(\Omega_j, \Omega_i)$, $c_i(\Omega_i) = c(\Omega_i)$, $m_i = m$, for all $i \in\{m,f\}$; considering a point on the diagonal $(\tilde{\Omega},\tilde{\Omega})$ with $\tilde{\Omega} \neq 0,1$, the marginal fitness function for participation of sex i is $\theta_i = 2\Big((1-s_i)+\big(1-s_j\big)\Big) r b(\tilde{\Omega},\tilde{\Omega}) - (1-(1-2\varpi M_i) r) c\big(\tilde{\Omega}\big)$. If $M_i < M_j$, then $\theta_i > \theta_j$ for every possible value of $\tilde{\Omega}$, and in this case selection will drive the population off of the diagonal, and specifically towards the sex-i-only equilibrium, which implies that the basin of attraction for the sex-i-only equilibrium encompasses the diagonal and is therefore larger than the basin of attraction for the equilibrium where only the other sex participates.

6 Illustrations: war outcome functions

For the purposes of illustration alone (Fig. 1,2) we make the war outcome functions explicit Notice that the results presented in this study and especially conditions (1-3) do not hinge on any particular functionalisation. We consider that the probability that the attacking group wins the war ω is a function of the fighting strengths σ_{att} and σ_{def} of the attacking and defending groups, specifically $\omega = \sigma_{att} / (\sigma_{att} + \sigma_{def})$. We consider that the fighting strengths of the two groups are themselves functions of a) the number of fighting individual of the two sexes, that is $N{\cdot}K{\cdot}\Omega_{i,att}$ and $N{\cdot}K{\cdot}\Omega_{i,def}$ (for the attacking and defending groups, respectively, assuming equal numbers of adults of the two sexes N_m=N_f=N) and b) the fighting effectiveness of the two sexes (ψ_i, for all $i\in\{m,f\}$). Considering that groups do not differ in total size and there are equal numbers of young adults of the two sexes and therefore only the proportions of fighting individuals of the two sexes influence the final outcome, we assume functional form $\sigma_{att} = ½ (\psi_i \Omega_{i,att} + \psi_j \Omega_{j,att})$ and $\sigma_{def} = ½ (\psi_i \Omega_{i,def} + \psi_j \Omega_{j,def})$.

7 Additional references

75. Fisher RA. 1930 *The Genetical Theory of Natural Selection*. Oxford, UK: The Clarendon Press.

76. Price GR, Smith CAB. 1972 Fisher's Malthusian parameter and reproductive value. *Ann. Hum. Genet.* **36,** 1-7.

77. Bulmer M (1994) *Theoretical Evolutionary Ecology*. Sunderland, MA: Sinauer Associates.

78. Grafen A. 2014 Total reproductive values for females and for males in sexual diploids are not equal. *J. Theor. Biol.* **359,** 233-235.
